# Significance of circulating microRNAs in diabetes mellitus type 2 and platelet reactivity: bioinformatic analysis and review

**DOI:** 10.1186/s12933-019-0918-x

**Published:** 2019-08-30

**Authors:** Justyna Pordzik, Daniel Jakubik, Joanna Jarosz-Popek, Zofia Wicik, Ceren Eyileten, Salvatore De Rosa, Ciro Indolfi, Jolanta M. Siller-Matula, Pamela Czajka, Marek Postula

**Affiliations:** 1 Department of Experimental and Clinical Pharmacology, Center for Preclinical Research and Technology CEPT, Medical University of Warsaw, Banacha 1B str., 02-097 Warsaw, Poland; 2 Rheumatology Division, Hospital das Clinicas HCFMUSP, University of São Paulo, School of Medicine, Av. Dr. Arnaldo, 455, Sao Paulo, SP 01246-903 Brazil; 3 Division of Cardiology, Department of Medical and Surgical Sciences, “Magna Graecia” University, Viale Europa, 88100 Catanzaro, Italy; 4 Department of Cardiology, Medical University of Vienna, Vienna, Austria

**Keywords:** MicroRNA, Diabetes mellitus type 2, Platelet reactivity, Biomarker, Bioinformatic analysis, miRNA–gene target interaction, Diagnosis, Prognosis

## Abstract

In the light of growing global epidemic of type 2 diabetes mellitus (T2DM), significant efforts are made to discover next-generation biomarkers for early detection of the disease. Multiple mechanisms including inflammatory response, abnormal insulin secretion and glucose metabolism contribute to the development of T2DM. Platelet activation, on the other hand, is known to be one of the underlying mechanisms of atherosclerosis, which is a common T2DM complication that frequently results in ischemic events at later stages of the disease. Available data suggest that platelets contain large amounts of microRNAs (miRNAs) that are found in circulating body fluids, including the blood. Since miRNAs have been illustrated to play an important role in metabolic homeostasis through regulation of multiple genes, they attracted substantial scientific interest as diagnostic and prognostic biomarkers in T2DM. Various miRNAs, as well as their target genes are implicated in the complex pathophysiology of T2DM. This article will first review the different miRNAs studied in the context of T2DM and platelet reactivity, and subsequently present original results from bioinformatic analyses of published reports, identifying a common gene (*PRKAR1A*) linked to glucose metabolism, blood coagulation and insulin signalling and targeted by miRNAs in T2DM. Moreover, miRNA–target gene interaction networks built upon Gene Ontology information from electronic databases were developed. According to our results, miR-30a-5p, miR-30d-5p and miR-30c-5p are the most widely regulated miRNAs across all specified ontologies, hence they are the most promising biomarkers of T2DM to be investigated in future clinical studies.

## Introduction

Type 2 diabetes mellitus (T2DM) is defined by chronic hyperglycemia and defective metabolism of carbohydrates, lipids, and proteins caused by inadequate insulin secretion and/or insulin action. The disease and its complications constitute a major health challenge. In line with the most recent reports by the International Diabetes Federation, nearly half a billion people across the globe live with diabetes, and epidemiological data predict an unsustainable increase in its prevalence in the upcoming years [1]. The worldwide epidemic of T2DM is mainly driven by the choice of lifestyle, physical inactivity, caloric excess, obesity, as well as aging and urbanization, however genetic component is also known to be involved in its pathogenesis [2, 3]. Furthermore, it is worth noticing that the epidemiology of diabetes has been changing over the past decades. The disease once thought to affect almost exclusively elderly population, is now often diagnosed among children and adolescents, hence placing a significant number of people at risk of developing serious complications and increasing the rates of diabetes-related morbidity [4]. Moreover, relative mortality rates in T2DM patients are also reported to be approximately 2- to 4-times as high compared with individuals without T2DM, and mostly linked to an increased risk of cardiovascular disease (CVD) [5]. Thus, the rapidly increasing incidence and prevalence of T2DM raises an urgent need to develop effective tools for early detection of the disease in order to prevent the progression of T2DM and improve patients’ clinical outcomes.

Platelets are thought to be important cellular components involved in both the initiation and progression of atherosclerosis, and even more so in the ensuing atherothrombotic sequelae [6]. Disturbances in the pro-inflammatory state and endothelial dysfunction observed in T2DM further contribute to the phenomenon of hypercoagulable state [7, 8]. The interplay of impaired insulin secretion and action, inflammatory process, defective glucose metabolism and platelet activation was put at the center stage to elucidate the pathophysiology of T2DM and its most common, cardiovascular complications [8–11]. The intensified adhesion, activation, aggregation, and platelet-derived thrombin generation are typical platelet alterations in T2DM platelets [12]. Our main focus was on the increased platelet activation in diabetic populations, which was reported in the past [8]. Platelets play a significant role in coordinating inflammation and immune responses, hence their increased activity may result in ameliorated inflammatory response [13]. Moreover, platelets release microparticles (MPs), which contribute to the modulation of immune cells function [14]. These molecules were formerly thought to derive from innate immune cells, including neutrophils and monocytes, however MPs originating from platelets are the most expressed in the circulation and deemed crucial in inflammation [15]. Combining the knowledge about these mechanisms and their main molecular components is the first step in discovering and designing novel biomarkers of T2DM and impaired platelet function.

MicroRNAs (miRNAs) due to their biochemical stability and abundance in different body fluids, emerged as potential novel biomarkers for T2DM and its complications. MicroRNAs are a class of small (18–25 nucleotides), endogenous, noncoding RNAs that regulate various aspects of cellular functions through suppression of gene expression [16, 17]. They are involved in a wide array of biological processes, such as cell development, proliferation, differentiation, as well as apoptosis or metabolism [18]. Until now, up to 2000 miRNAs have been listed for *Homo sapiens*, and it is estimated that the majority of protein-coding genes are directly regulated by these molecules. What is more, each miRNA can bind and regulate more than one target, exerting its effects on several levels simultaneously. Although some circulating miRNAs exhibit low expression, there is evidence of considerable redundancy among the targets. The same UTRs are targeted by numerous miRNAs thus even low expressed miRNAs exert their functions as part of an epigenetic regulation network. These miRNAs are fine-tuners of gene expression patterns in response to pathophysiological stimuli and despite being expressed at relatively low levels under basal conditions, miRNAs are strongly up-regulated during pathological stress [19]. Even though the majority of miRNAs are found intracellularly, a significant amount appears in the extracellular space, including the blood and other body fluids [20]. It is worth mentioning that platelets harbor large amounts of miRNAs. Consequently, they are the major source for circulating miRNAs, with a relevant regulatory potential on cardiovascular pathophysiology [21]. Moreover, circulating miRNAs correspond with the amount of platelet microvesicles, which are also abundantly released into circulation [21]. Thanks to their functional role and increasingly advanced detection technologies, circulating miRNAs can be extracted from plasma, serum, or whole blood, and studied in the context of becoming novel diagnostic and prognostic disease signatures.

Up to date, multiple methods were proposed and applied by researchers to detect and investigate miRNAs, including northern blotting, quantitative reverse transcription polymerase chain reaction (qRT-PCR) and microarrays. All mentioned methods have their advantages and limitations. Northern blot technology was commonly used owing to its high specificity and ability to utilize sequences with even partial homology as hybridization probes [22]. On the other hand, low detection efficiency, contamination due to radiolabeling, and poor sensitivity for oligonucleotide probes might lessen the value of northern analysis measurements [23]. Currently, qRT-PCR and microarrays are the most popular techniques for reliable and effective detection of miRNAs. While northern blot and qRT-PCR enable the analysis one by one, microarray offers to test a few hundred miRNAs per single run [24]. Along with the growing interest in miRNA use in diagnostic and therapeutic strategies, RT-PCR innovations are being proposed to enhance its efficiency. For instance, integrating with microfluidic filtering devices, as well as digital droplet PCR, are new improvements that not only offer higher analytical standards, but also require minimal reaction volumes [25]. Furthermore, next-generation sequencing (NGS) merits mention as a newly preferred technique for studying circulating miRNAs. Its main advantage is the possibility to sequence samples simultaneously by pooling with NGS and to build expression profiles for every investigated sample. The use of NGS is often limited by high cost, time consumption and staff involvement [26, 27]. Alternative techniques are also being developed, among them the use of nanosensors or nanowires, or the enzyme-linked assays that enable miRNAs measurement through direct hybridization [28].

Herein, we present an up-to-date review on diagnostic and prognostic value of miRNAs related to T2DM and processes potentially related to platelet reactivity based on human studies and report the results of a quantitative bioinformatic analysis highlighting the most promising miRNAs for clinical application in T2DM. Since the pathogenesis and complications of T2DM are very complex, the aim of our bioinformatic analysis was to search for common miRNAs related to processes such as glucose metabolism, inflammation or blood coagulation that can potentially be linked to platelet activation pathways. We believe that the results of our bioinformatic analysis, which revealed novel miRNA targets, could be further validated in laboratory and clinical settings and help to create a paradigm for future studies in this field.

### Article search process

Electronic database Pubmed and Scopus was searched between July 2018 and November 2018, and original studies were reviewed to evaluate the potential diagnostic or/and prognostic role of circulating miRNAs associated with T2DM. Review articles and meta-analyses were also investigated, and their secondary references were examined for possible inclusion. The following search syntax was used: “Search (“MicroRNAs” [MeSH Terms] OR “mir” [MeSH Terms] OR “mirna” [MeSH Terms] OR “circulating miRNA” [MeSH Terms] OR “circulating microRNA” [MeSH Terms]) AND (“diabetes mellitus, type 2” [Mesh] [MeSH Terms] OR “T2DM” [All Fields]) AND (“diagnostic” [MeSH Terms] OR “diagnosis” [All Fields]) OR (“prognostic” [MeSH Terms] OR “prognosis” [All Fields]) AND (“blood platelets” [MeSH Terms] OR “Platelet Activation” [MeSH Terms] OR “Platelet Aggregation” [MeSH Terms] OR “Platelet reactivity” [All Fields])” Filters: Humans. Our search was limited to human studies and did not exclude studies on the basis of ethnicity of study participants. Titles and abstracts were screened by two independent operators.

### Circulating miRNAs and T2DM

In light of emerging reports, differential concentration of circulating miRNAs could offer promising opportunities for diagnosis, prognosis and treatment monitoring in T2DM. The number of studies investigating the molecules’ characteristics in the context of T2DM is constantly increasing, however available evidence is still limited and should be further expanded in order to elucidate the complex interactions between miRNAs and other molecules relevant in T2DM pathophysiology, as well as their potential use in clinical practice. In this section we provide a description and summary of the studies that investigated various circulating miRNAs in T2DM (see Tables 1, 2; Additional file 1).

**Table 1:** Characteristics of microRNA studies in T2DM

First author (year of publication) [reference]	Studied miRNAs	Expression of miRNAs	T2DM/controls	Method	Mechanism
Kong (2011) [29]	miR-9 miR-29a miR-30d miR-34a miR-124a miR-146a miR-375	↑ miR-9 ↑ miR-29a ↑ miR-30d ↑ miR-34a ↑ miR-124a ↑ miR-146a ↑ miR-375	18/19	qRT-PCR	Glucose metabolism
Karolina (2012) [30]	miR-27a miR-320a	↑ miR-27a ↑ miR-320a	50/46	microRNA profiling And qRT-PCR	Glucose metabolism
Zhang (2013) [31]	miR-29b miR-15a miR-28-3p miR-223 miR-126	↓ miR-126	30/30	qRT-PCR	Glucose metabolism
Liu (2014) [32]	miR-126	↓ miR-126	160/138	qRT-PCR	Glucose metabolism
Ghorbani (2017) [33]	miR-21 miR-126 miR-146a	↓ miR-21	45/42	qRT-PCR	Glucose metabolism
Al-Muhtaresh (2018) [34]	miR-375 miR-9	↑ miR-375 ↑ miR-9	30/30	qRT-PCR	Glucose metabolism
Jiménez-Lucena (2018) [35]	miR-103 MiR-107 miR-126 miR-143 miR-144 miR-145 miR-150 miR-15a miR-182 miR-192 miR-21 miR-223 miR-28-3p miR-29a miR-30a-5p miR-30d miR-320 miR-33a miR-375 miR-657 miR-7 miR-9 miR-96	↑ miR-30a-5p ↑ miR-150 ↓ miR-103 ↓ miR-28-3p ↓ miR-29a ↓ miR-9 ↓ miR-15a ↓ miR-126 ↓ miR-145 ↓ miR-375 ↓ miR-223	107/355	qRT-PCR	Glucose metabolism
Zampetaki (2010) [36]	miR-24 miR-21 miR-20b miR-15a miR-126 miR-191 miR-197 miR-223 miR-320 miR-486 miR-150 miR-29b miR-28-3p	↑ miR-28-3p **↓** miR-24 **↓** miR-21 **↓** miR-20b **↓** miR-15a **↓** miR-126 **↓** miR-191 **↓** miR-197 **↓** miR-223 **↓** miR-320 **↓** miR-486 **↓** miR-150 **↓** miR-29b	80/80	microRNA profiling and qRT-PCR	miR-126: endothelial dysfunction
Zhang (2015) [37]	miR-126	↓ miR-126	20/20	qRT-PCR	Glucose metabolism
Balasubramanyam (2011) [44]	miR-146a	↓ miR-146a	20/20	qRT-PCR	Inflammation
Luo (2015) [45]	miR-103b	↓ miR-103b	79/46	qRT-PCR	Inflammation
Olivieri (2015) [47]	miR-126-3p miR-21-5p	↓ miR-126-3p ↓ miR-21-5p	193/107	qRT-PCR	Inflammation
Giannella (2017) [48]	miR-126-3p miR-126-5p	↓ miR-126-3p	68/53	qRT-PCR	Inflammation
Witkowski [49]	miR-126	↓ miR-126	46/-	QRT-PCR	Inflammation
Jansen (2016) [54]	miR-126 miR-222 miR-let7d miR-21 miR-30 miR-92a miR-139 miR-199a MiR-26a	↓ miR-126 ↓ miR-26a	55/80	qRT-PCR	Endothelial dysfunction
Deng (2017) [55]	miR-24	↓ miR-24	28/31	qRT-PCR	Endothelial dysfunction
Amr (2018) [56]	miR-126 MiR-210	↓ miR-126 ↑ miR-210	100/20	qRT-PCR	miR-126: Endothelial dysfunction miR-2010: hypoxia
Stępień (2018) [57]	miR-126-3p miR-126-5p miR-193b-3p miR-199a-3p miR-20a-3p miR-221-3p miR-23b-3p miR-26a-5p miR-26b-5p miR-29a-5p MiR-30b-5p miR-30c-5p miR-374a-5p miR-409-3p miR-495-3p miR-95-3p let-7i-5p	↑ miR-193b-3p ↑ let-7i-5p ↑ miR-199a-3-5p ↑ miR-26b-5p ↑ miR-30b-5p ↑ miR-374a-5p ↑ miR-20a-3p ↑ miR-26a-5p ↑ miR-30c-5p ↓ miR-409-3p ↓ miR-95-3p	15/15	microRNA profiling and qRT-PCR	Angiogenesis
Stratz (2014) [59]	miR-377-5p miR-628-3p miR-3137	No significant differences in platelet miRNA profiles	30/30	microRNA profiling and qRT-PCR	Platelet reactivity
Fejes (2017) [60]	miR-223 miR-26b miR-126 MiR-140	↓ miR-223 ↓ miR-26b ↓ miR-126 ↓ miR-140	28/23	qRT-PCR	Platelet reactivity

**Table 2:** Expression and statistical analysis results of microRNAs studied in T2DM

First author [reference]	Expression of miRNA	OR	p value	AUC	
				Sensitivity	Specificity
Kong (2011) [29]	↑ miR-9 ↑ miR-29a ↑ miR-30d ↑ miR34a ↑ miR-124a ↑ miR146a ↑ miR-375		0.021	–	–
			0.003		
			0.12		
			0.001		
			0.12		
			0.01		
			0.002		
Karolina (2012) [30]	↑ miR-27a ↑ miR-320a	–	0.010 0.019	–	–
Zhang (2013) [31]	↓ miR-126	–	< 0.01	–	–
Liu (2014) [32]	↓ miR-126	3.5	< 0.05	0.792	–
Ghorbani (2017) [33]	↓ miR-21	–	0.01/0.03	–	–
Al-Muhtaresh (2018) [34]	↑ miR-375 ↑ miR-9	1.12–1.151 0.972–1.006	0.001–0.05 0.33–0.954	0.78 0.532	–
Jiménez-Lucena (2018) [35]	↑ miR-30a-5p ↑ miR-150 ↓ miR-103 ↓ miR-28-3p ↓ miR-29a ↓ miR-9	–	< 0.001 < 0.001 < 0.001 < 0.001 < 0.001 < 0.001	–	–
Zampetaki (2010) [36]	↑ miR-28-3p **↓** miR-24 **↓** miR-21 **↓** miR-20b **↓** miR-15a **↓** miR-126 **↓** miR-191 **↓** miR-197 **↓** miR-223 **↓** miR-320 **↓** miR-486 **↓** miR-150 **↓** miR-29b	–	–	–	–
Zhang (2015) [37]	↓ miR-126	0.967	0.0158	0.7778	0.6667
Balasubramanyam (2011) [44]	↓ miR-146a	–	0.015	–	–
Luo (2015) [45]	↓ miR-103b	–	< 0.05	–	–
Olivieri (2015) [47]	↓ miR-126-3p ↓ miR-21-5p	–	0.032 < 0.001	–	–
Giannella (2017) [48]	↓ miR-126-3p	–	0.001, < 0.001	–	–
Witkowski [49]	↓ miR-126	–	0.226	–	–
Jansen [54]	↓ miR-126 ↓ miR-26a	–	0.226 0.0094	–	–
Deng (2017) [55]	↓ miR-24	–	< 0.01	0.975	
Amr (2018) [56]	↓ miR-126 ↑ miR-210		< 0.01 < 0.01	0.96–0.98 0.95–0.98	–
Stępień (2018) [57]	↑ miR-193b-3p ↑ let-7i-5p ↑ miR-199a-3-5p ↑ miR-26b-5p ↑ miR-30b-5p ↑ miR-374a-5p ↑ miR-20a-3p ↑ miR-26a-5p ↑ miR-30c-5p ↓ miR-409-3p ↓ miR-95-3p	–	0.015 0.006 0.001 0.012 0.01 0.00001 0.064 0.075 0.055 0.004 0.041	–	–
Stratz (2014) [59]	No significant differences in platelet miRNA profiles				
Fejes (2017) [60]	↓ miR-223 ↓ miR-26b ↓ miR-126 ↓ miR-140	–	< 0.01/0.382 < 0.01/0.011 – –	–	–

### MicroRNA studies linked with glucose metabolism and development of diabetes

The development of T2DM and resulting hyperglycemia have several underlying mechanisms. Insulin is the key hormone responsible for glucose homeostasis. T2DM subjects manifest abnormalities both in tissue sensitivity to insulin, as well as in pancreatic insulin secretion [3]. In healthy individuals, pancreatic beta cells conform to changes in insulin action, hence a potential decrease in insulin action is followed by intensified insulin secretion and vice versa. Any alteration of this dynamic interaction leads to dysfunctional glucose metabolism [3]. Whereas the studies below describe the potential relationship between miRNAs and glucose metabolism, other contributing mechanisms such as inflammation and platelet activation should be taken into account while analyzing the research outcomes.

In one of the initial studies conducted to deepen the understanding of glucose metabolism in T2DM the expression profiles of several miRNAs relevant to the pathogenesis of T2DM were investigated and compared among three subgroups of patients enrolled in the study: patients with newly diagnosed T2DM, pre-T2DM subjects and T2DM-susceptible subjects with normal glucose tolerance (NGT) [29]. The authors reported significantly higher expression of all analyzed miRNAs in T2DM patients compared to T2DM-susceptible subjects. Moreover, certain miRNAs were significantly down-regulated in the pre-T2DM individuals vs. T2DM patients. Interestingly, the expression patterns of circulating miRNAs in pre-T2DM group were similar to the ones observed in patients with NGT. On one hand, this study strengthens the hypothesis that miR-9, miR-29a, miR-30d, miR-34a, miR-124a, miR146a and miR-375 may play a role in the regulation of insulin and pathogenesis of T2DM. On the other hand, their expression levels do not change dramatically in pre-T2DM stage, which undermines their usefulness as a disease-specific biomarker [29].

On the other hand, Karolina et al. aimed to identify miRNAs of patients with metabolic syndrome and compare them with subjects manifesting T2DM, among other metabolic traits. The study population comprised of five different subgroups, including metabolic syndrome subjects, T2DM subjects, hypercholesterolemic subjects, hypertensive subjects and healthy controls [30]. As far as T2DM was concerned, an association was found between miR-27a and miR-320a and metabolic syndrome. MicroRNA-150, miR-192, miR-27a, miR-320a, and miR-375 were up-regulated in T2DM, which supports the hypothesis that they might be involved in the regulation of hyperglycemia and should be investigated in greater depth. The current findings indicate a potential clinical use of these miRNAs in estimation of risk of T2DM and metabolic syndrome [30].

Also, Zhang et al. [31] conducted a cross-sectional study to investigate the miRNA profile among individuals with appropriate glycemia, individuals susceptible of developing T2DM and T2DM patients, and aimed to estimate their predictive value. The expression of several circulating miRNAs was compared between the different groups, however only miR-15a, miR-223 and miR-126 were detectable in plasma. Interestingly, plasma miR-126 levels were analogous between pre-T2DM and T2DM groups, yet substantially lower than in NGT group. Furthermore, the expression pattern of plasma miR-126 was linked with fasting glucose levels. The study revealed that both detection and estimation of plasma miR-126 level could be potentially considered as a non-invasive diagnostic tool to predict and prevent T2DM [31].

The use of differential expression of circulating miR-126 as potential biomarker of pre-T2DM and T2DM was further investigated by Liu et al. [32]. The analysis was performed among impaired glucose tolerance (IGT) subjects, impaired fasting glucose (IFG) subjects, recently diagnosed T2DM patients as well as healthy individuals, and demonstrated that substantially lower values of miR-126 characterized T2DM and impaired IGT/IFG subjects in comparison to healthy individuals. Additionally, receiver operator characteristic (ROC) analysis of serum miR-126 allowed to differentiate T2DM/IFG/IGT patients from control subjects. The study was not only extended to include pre-T2DM patients but also evaluated a potential therapeutic response value of miR-126. It was reported that serum miR-126 increased after 6 months of diet control and physical exercise in IGT/IFG subjects, as well as 6 months of diet control, exercise and insulin treatment in T2DM patients. The findings show the likely use of miR-126 to both effectively diagnose T2DM at early stages and monitor the therapeutic response [32].

Furthermore, serum miRNAs were recently evaluated in T2DM patients and control subjects in order to find the potential relationship between these miRNAs, obesity and T2DM [33]. In contrast to previous reports, no significant difference in the expression of the above-mentioned miRNAs was observed between the studied cohorts. Although miR-21 was significantly down-regulated in both obese T2DM, as well as obese non-T2DM patients compared to lean individuals, miR-126 and miR-146a did not correlate with biochemical parameters in the patient and control groups. The study demonstrates that serum miR-21 expression does not correlate with T2DM, nonetheless it correlates negatively with obesity in both T2DM and non-T2DM groups. Additionally, miR-21 level is linked with clinical and biochemical metabolic patterns characteristic for obesity, namely BMI and waist circumference, in the evaluated cohorts [33].

The diagnostic value of miR-375 and miR-9, known to influence the regulation of insulin secretion, was investigated in early detection of pre-T2DM states and T2DM among three different cohorts consisting of pre-T2DM patients, T2DM patients and controls [34]. The expression of both circulating miRNAs was elevated in pre-T2DM and T2DM groups compared to controls. Furthermore, miR-375 and miR-9 were found to be directly linked to the presence of pre-T2DM and T2DM independently of risk factors, whereas miR-375 was independently associated with the development of T2DM. In line with ROC analysis results, miR-375 is suitable for differentiating T2DM patients from healthy individuals, as well as pre-T2DM from T2DM subjects, although miR-9 offers borderline significance in differentiating the patient cohorts. It is worth noticing that it is the combination of both miRNAs that showed improved predictability to discriminate the patients with inappropriate glycemia from control subjects. The findings indicate that miR-375 and miR-9 correlate with the susceptibility to developing T2DM and should be further studied to confirm their potential biomarker characteristics [34].

More recently, Jiménez-Lucena et al. [35] analyzed plasma miRNAs related to insulin sensitivity, secretion and growth among subjects with incidentally developed T2DM and non-T2DM controls. As far as baseline plasma levels were concerned, nine miRNAs (miR-9, miR-15a, miR-28-3p, miR-29a, miR-103, miR-223, miR-126, miR-145, and miR-375) were significantly down-regulated, whereas two miRNAs (miR-30a-5p and miR-150) were significantly up-regulated in incident-T2DM vs. non-T2DM subjects. In line with the ROC analysis results, miR-9, miR-28-3p, miR-29a, miR-30a-5p, miR-103, miR-126, miR-150, miR-223, and miR-375, combined with glycated hemoglobin (HbA1c), have a higher diagnostic value in T2DM than HbA1c alone. Finally, Cox regression analysis demonstrated that patients with down-regulated miR-103, miR-28-3p, miR-29a, and miR-9 and up-regulated miR-30a-5p and miR-150 are more likely to develop T2DM. To conclude, the findings support the hypothesis that miRNAs could serve as a diagnostic tool to predict the development of T2DM [35].

In one of the prospective studies using microarray methodology the potential utility of miRNAs as diagnostic biomarkers was evaluated in T2DM patients and matching controls [36]. It was revealed that miR-20b, miR-21, miR-24, miR-15a, miR-126, miR-191, miR-197, miR-223 and miR-320 were down-regulated, whereas miR-486 and miR-28-3p were up-regulated in prevalent T2DM. On the other hand, down-regulation of miR-15a, miR-29b, miR-126, miR-223, and up-regulation of miR-28-3p negatively correlated with the manifestation of T2DM. Interestingly, miR-126 emerged as a dynamic and significant diagnostic tool of manifest T2DM, hence its expression was evaluated in the entire Bruneck cohort and assessed in both univariate and multivariate analyses. The authors further reported that the content of miR-126 in apoptotic bodies of exosomes and circulating vesicles in plasma is diminished in high glycemia. Overall, Zampetaki et al. [36] presented a miRNA profile characteristic for T2DM, which partially explains the change in angiogenic status among these patients.

The diagnostic role of miR-126 in predicting the onset of T2DM was reported in another prospective study conducted in groups of T2DM patients and healthy controls, whose authors found that the baseline levels of miR-126 were substantially lower among subjects who developed T2DM in 2 years compared to individuals with NGT [37]. The results revealed that miR-126 might correlate with onset of T2DM, and if more investigations on larger populations confirm these findings, this miRNA could be applied as a tool to detect the T2DM before its clinical manifestations emerge [37].

The studies described above suggest that miRNAs are differentially expressed among T2DM-susceptible subjects, T2DM patients and healthy individuals and indeed have a diagnostic potential. Glucose metabolism dysfunction has a significant impact on other cells, tissues and processes, which altogether give rise to T2DM. However, identifying the various molecules which take part in individual pathways, might aid in designing new diagnostic tools. As observed, miR-126 and miR-375 are among the most commonly reported miRNAs in these studies, hence efforts should be made to elucidate their role in T2DM pathogenesis and potential use in diagnostic and therapeutic strategies. It is worth noticing that the study by Zampetaki et al., as well as by Zhang et al. provided prospective data in terms of biomarker potential of microRNAs for the onset of T2DM, in contrast to other cross-sectional studies, which have numerous limitations [36, 37]. Cross-sectional data fail to accurately establish causal order, in particular at the level of an individual, and cannot be used to analyze the variations of miRNA expression over a period of time. These types of bias are essential to consider while analyzing the studies.

### MicroRNA studies linked with inflammation

Evidence linking systemic inflammation to T2DM has been continuously accumulating. Whereas the triggering mechanisms of inflammation in T2DM pathogenesis are still ill-understood, the implications of chronic activation of proinflammatory pathways in target cells of insulin action likely contribute to various metabolic disorders, including T2DM. The relationship is further strengthened by the presence of key inflammatory biomarkers, such as leptin, tumor necrosis factors (TNFs), interleukin-6 (IL-6), C–C motif chemokine 2, resistin or adiponectin, in the event of T2DM and complications [12, 38–40]. Moreover, it was reported that platelet hyperreactivity in T2DM can occur in response to systemic inflammation, as evidenced by the increased expression of platelet FcgammaRIIA receptor, an immunoreceptor on the platelet surface that has been shown to activate platelets through a common signaling pathway that includes the tyrosine kinase Syk and phospholipase Cγ2 [41–43]. Higher expression of platelet FcgammaRIIA is observed in patients with T2DM, as well as acute coronary or cerebrovascular events, among others. These findings led to speculations that increased platelet FcgammaRIIA predisposes to occurrence of atherothrombotic events [43]. Since inflammation and platelet reactivity are largely intertwined, the studies presented below could share elements of both mechanisms.

The role of miR-146a expression along with its downstream proinflammatory signals in relation to glycemia and insulin resistance was evaluated by Balasubramanyam et al. [44] in patients with T2DM and compared to NGT controls. In line with obtained results, the expression level of miR-146a was significantly decreased in T2DM patients vs. control subjects. Furthermore, the authors studied the expression of two confirmed gene targets of miR-146a, namely *TRAF6* and *IRAK1* along with nuclear factor κΒ (NFκβ) messenger RNA (mRNA) levels and found that the *TRAF6* expression was significantly increased in patients with T2DM compared to controls, whereas no significant difference was seen in the expression of NFκβ and *IRAK1*. The findings revealed that miR-146a expression negatively correlated with fasting blood glucose (FBG), HbA1c, insulin resistance, proinflammatory signals such as *TRAF6* and NFκβ mRNA levels and also concentration of proinflammatory cytokines TNFα and IL-6. Overall, it was reported that an impairment and down-regulation of miR-146a correlates with inflammatory changes and insulin resistance observed in T2DM [44].

Moreover, the expression profile of platelet-derived miR-103b, a regulator of secreted frizzled-related protein 4 (SFRP4), was studied to determine whether miR-103b can be used as a potential biomarker for early detection of T2DM [45]. Four groups of participants were evaluated in the study, namely healthy subjects, pre-T2DM subjects, non-complicated T2DM subjects and T2DM-coronary heart disease (CHD) subjects. The correlation between miR-103b levels and its target gene *SFRP4* expression was also investigated. The *SFRP4* is involved in inflammatory processes and was formerly found to be over-expressed in the pancreatic islets of T2DM patients, suggesting its promising use as a biomarker of T2DM [46]. The results showed that miR-103b expression was significantly decreased in pre-T2DM compared to the control subjects, whereas the expression of the *SFRP4* gene was increased in platelets and pre-T2DM subjects. Interestingly, platelet-derived miR-103b was down-regulated and *SFRP4* up-regulated in patients subjected to antiplatelet treatment with acetylsalicylic acid, adding a potential therapeutic response value to its use as a biomarker. In line with these findings, miR-103b might have a negative regulatory function in the expression of SFRP4 mRNA/protein in pre-T2DM, and hence correlate with the early development of T2DM [45].

MicroRNAs linked to endothelial function and inflammatory processes in T2DM, miR-126-3p and miR-21-5p respectively, were also evaluated among four different groups of patients—healthy controls, T2DM subjects, T2DM subjects without (T2DM NC) and with (T2DM C) complications [46]. The expression of miR-126-3p and miR-21-5p decreased significantly from controls to T2DM NC and T2DM C, nevertheless the differences in miRNA expression were not significant between patients without and with T2DM complications. It is worth mentioning that out of all studied T2DM complications, the levels of both miRNAs differed significantly only between T2DM subjects with and without the history of major cardiovascular events (MACE). In line with multivariate analysis results, miR-21-5p and miR-126-3p were esteemed significant variables in the development of MACE complications. Overall, both miR-21-5p and miR-126-3p were found to contribute to the inflammatory and endothelial dysfunction in T2DM patients, predicting their future use in estimating the risk of occurrence of T2DM complications [47]. It merits special mention that inflammation and increased platelet activation are closely linked to each other and contribute to endothelial dysfunction, as well as resulting vascular changes in T2DM.

Recently, the level of circulating MPs, their content of miR-126-3p and miR-126-5p and association with endothelial activation and dysfunction was studied in patients with various levels of inappropriate glycemia [48]. Subjects with IGT, T2DM patients and healthy controls were included in the study. Firstly, the authors noticed that only CD62E^+^ MPs was significantly higher among pre-T2DM and T2DM patients in comparison to glucose tolerant individuals. Multiple linear analysis demonstrated that only plasma glucose degree was the most independent forecaster of CD62E^+^. Moreover, miR-126-3p was shown to be was substantially higher in MPs among healthy individuals in contrast to comparable expression between patients with pre-T2DM and T2DM, and no significant correlation was found between miR-126-5p and the rest of subjects. Overall, the levels of circulating CD62E^+^ MPs and miR-126-3p contained in MPs are different in subjects with various level of glycemia and miR-126-3p value is associated with generally approved endothelial dysfunction markers such as vascular cell adhesion protein-1 (VCAM-1), plasma antioxidant level and MPs [48].

Interestingly, miR-126 level was also shown to correlate with therapeutic response in T2DM, hence it is vital to consider antidiabetic treatment while interpreting its expression profiles. Up-regulation of miR-126 in the circumstances of uncontrolled T2DM was found by Witkowski et al. [49], who evaluated the contribution of miRNA to circulating tissue factor (TF) expression and thrombogenicity in T2DM patients before and after optimization of antidiabetic treatment. They reported that low miR-126 was linked to increased TF, TF-mediated thrombogenicity, as well as increased vascular inflammation. Furthermore, miR-126 levels increased and thrombogenicity was reduced once antidiabetic treatment had been optimized, hence suggesting the antithrombotic properties of miR-126. The studies by Witkowski et al., as well as by Liu et al., show that miR-126 levels might be restored by effective antidiabetic treatment and dietary interventions, and be applied as a marker of therapeutic response [32, 49]. The study also reflects the regulation pattern characteristic for miR-126, which appears to be one of the main miRNAs responding to diabetic environment. It was found that the expression of miR-126 is regulated by transcription factors Ets-1 and Ets-2 [50]. Witkowski et al. reported that high-glucose treatment of endothelial cells decreased ets-1 expression, which suggests that ect-1 might be glucose-sensitive [49]. On the other hand, the miR-126 is down-regulated by hyperglycemia/vascular inflammation, as evidenced by proinflammatory cytokine TNFα, elevated leukocyte count and fibrinogen levels [49].

The evidence reviewed above indicate that inflammation is a characteristic feature of T2DM and should be considered while designing further studies aimed at evaluating the clinical use of miRNAs in T2DM. As it was described above, some miRNAs are down-regulated via pro-inflammatory cytokines, such as TNFa, which was shown to hamper miR-126 expression [49]. High miR-126 expression was found to correlate negatively with markers of vascular inflammation such as leukocyte count, fibrinogen levels, VCAM-1, endothelin and E-selectin. On the other hand, loss of anti-inflammatory miRNAs, including miR-126, increases vascular inflammation in T2DM, as evidenced by elevated leukocyte count and fibrinogen. As a result of VCAM1 3′UTR being directly targeted by miR-126, increased adhesion and transmigration of peripheral blood mononuclear cells takes place [49]. Once again, the involvement of miR-126 emerges as promising in T2DM [47, 48], however abnormal expression of miR-146a, miR-103b and miR-21 was also linked to inflammatory response and T2DM [44, 45, 47].

### MicroRNA studies linked with T2DM-associated vascular complications

Endothelial damage is well recognized in T2DM and defined as an imbalance in the production of vasodilator and vasoconstricting factors, predisposing the vasculature towards pro-thrombotic and pro-atherogenic effects [51]. Together with the evidence of hyperglycemia- and inflammation-induced abnormal platelet function, insights into endothelial dysfunction lead to a hypothesis of the etiology and pathogenesis of T2DM and its complications [52]. In fact, platelets are crucial in maintenance of appropriate vascular integrity. These cellular components are known to support the function of vascular endothelium, encourage growth of endothelial cells and release molecules, which improve endothelial barrier function. In response to inflammation, up-regulation of fibrinogen causes recruitment of inflammatory cells and platelets, as well as activation of endothelial cells, hence contributing to vascular inflammation [53]. Therefore, comprehension of mechanisms of both endothelial and platelet dysfunction, contributing to pathogenesis of T2DM, is deemed necessary to refine the vascular complications, frequently resulting in prothrombotic state [52].

In one of the studies vascular cells-expressed and MPs-derived miRNAs were investigated to determine their potential contribution to the pathogenesis of vascular complications in T2DM subjects and non-T2DM controls [54]. The expression profile of miR-26a and miR-126 differed significantly between patients with and without T2DM. Both miRNAs were found to be down-regulated in T2DM patients compared to controls. Interestingly, patients with down-regulated miR-26a and miR-126 were more likely to suffer from concomitant coronary artery disease (CAD), but not other comorbidities. Moreover, the authors demonstrated that the miRNAs were abundantly sourced from endothelial cells and by conducting further in vitro experiments, showed that high blood glucose levels contribute to diminished packaging of miR-126 and miR-26a into endothelial MPs. The study reveals that the expression profile of endothelial MPs-derived miRNAs is affected by T2DM and might be used to estimate the risk of potential vascular complications in this population [54].

Moreover, Deng et al. [55] evaluated the expression pattern of blood plasma-derived circulating miR-24 and its target YKL-40, which is an inflammatory molecule known to play an important role in endothelial dysfunction, in CHD patients, T2DM-CHD patients and controls. MiR-24 was significantly down-regulated in T2DM-CHD patients in contrast to control subjects. What is more, the expression of YKL-40 mRNA was remarkably increased in both T2DM-CHD and CHD patients vs. controls; as well as significantly higher in T2DM-CHD subjects compared to CHD subjects alone. The investigation of miR-24 as a potential biomarker was further analyzed with the help of ROC curve, which demonstrated significant ability to differentiate T2DM-CHD from CHD subjects and controls with an area under the curve (AUC) of 0.975, as well as discriminate T2DM-CHD from CHD subjects with an AUC of 0.953. These findings suggest that circulating miR-24 emerges as a potent regulator of YKL-40 and dynamic biomarker in predicting T2DM and development of cardiovascular complications [55].

Plasma miR-126 and miR-210 were also evaluated in the context of potential association between the miRNAs, T2DM and vascular complications in T2DM patients and healthy controls [56]. The expression of miR-126 was significantly reduced in T2DM subjects with and without CAD, respectively, in contrast to healthy controls. Interestingly, patients with CAD had lower expression of miR-126 compared to patients without CAD. On the other hand, miR-210 was significantly up-regulated by in T2DM subjects with and without CAD, respectively, in contrast to controls, as well as and in patients with CAD in contrast to those without CAD. It is worth noticing that in subjects with CAD, the levels of both miRNAs correlated with glycemia, HbA1c and lipid profile. Furthermore, the ROC curves revealed that plasma miR-126 and miR-210 significantly differentiated T2DM with CAD from T2DM without CAD. Overall, plasma miR-126 and miR-210 levels could be considered as epigenetic biomarkers for T2DM and vascular complications, especially CAD [56].

Most recently, angiogenic potential of ectosome-derived miRNAs was assessed among T2DM patients and controls [57]. The authors reported that 10 miRNAs (miR-20a-3p, miR-26a-5p, miR-26b-5p, miR-29a-5p, miR-374a-5p, miR-30b-5p, miR-30c-5p, let-7i-5p, miR-199a-3p, miR-221-3p) were found exclusively in ectosomes obtained from T2DM patients, as determined by low-density qPCR array. Moreover, it was revealed that the expression of both miR-193b-3p and miR-95-3p in the ectosomes-enriched plasma was significantly higher in T2DM, whereas the expression of miR-409-3p expression was lower in T2DM. Bioinformatics tools were then applied to determine the pro- and anti-angiogenic potential of these miRNAs. The analysis demonstrated that miRNAs from miR-26 family, as well as miR-221-3p have anti-angiogenic effects, while miRNAs from miR-30 family and miR-199a-3p have pro-angiogenic effects. Overall, the findings suggest a characteristic ectosome-specific miRNA content in diabetic population and may partially elucidate the phenomenon of impaired angiogenesis and development of vascular complications in patients with T2DM [57].

The above-mentioned studies showed correlations between various miRNAs, T2DM and vascular abnormalities. Among the others, it is worth mentioning that miR-126 was suggested as the miRNA that differentiates diabetic from non-diabetic populations.

### MicroRNA studies linked with platelet reactivity

Diabetic subjects are known to exhibit increased platelet activity [41, 53, 58]. Among the interrelated variables that contribute to this phenomenon are hyperglycemia, hyperlipidemia, oxidative stress, glycation of platelet membrane proteins, as well as impaired calcium homeostasis that lead to augmented platelet sensitivity to agonists [53]. Moreover, T2DM is often manifested by hypercoagulable state, known to be a result of disturbances in platelet activation, aggregation and lifespan, in addition to the pro-inflammatory state and endothelial dysfunction [12]. Elucidating the exact mechanisms and participating molecules would help identify T2DM patients at risk of cardiovascular complications and facilitate appropriate treatment.

One of the clinical studies evaluating miRNA signatures of platelets in patients with T2DM that are not linked to CAD was conducted by Stratz et al. [59]. MicroRNA was subjected to miRNA profiling, followed by qRT-PCR validation in both T2DM and non-T2DM patients. Contrary to previous reports, no miRNAs were significantly different between the studied groups, neither T2DM vs. non-T2DM, nor CAD vs. no-CAD, in univariate analyses. However, miR-377-5p, miR-628-3p, miR-3137 could be potential stable predictors of group membership. Functional network analysis of predicted targets for the investigated miRNAs suggest potential contribution of T2DM on mRNA processing. In conclusion, no significant differences were found in platelet miRNA expression between the T2DM cohort and controls, nonetheless functional implications of these molecules on mRNA processing can be important in the pathogenesis of T2DM. It is necessary to design and perform further studies to illustrate the functional network and fully elucidate the relations between miRNAs and T2DM [59].

On the other hand, Fejes et al. analyzed the effect of hyperglycemia on the expression of several platelet and circulating miRNAs derived from megakaryocytes (MKs) in T2DM obese patients, non-T2DM obese patients and healthy individuals [50]. Real-time PCR was used to quantify miRNA, as well as pre-miRNAs and their target mRNA, namely P2RY12 and SELP. It was reported that the expression of mature platelet miR-223, miR-26b, miR-140 and miR-126 was lower among T2DM subjects compared with healthy individuals. Circulating miR-223, miR-26b, miR-140 and miR-126 isolated from blood plasma were down-regulated in T2DM in similar fashion to platelet miRNAs. Furthermore, cell culture studies were performed to evaluate the influence of hyperglycaemia on the expression of miRNAs in MKs and revealed significantly reduced expression in the event of higher blood glucose levels. As far as platelet activation is concerned, significantly higher level of surface P-selectin expression was found in T2DM patients compared to healthy and obese controls, suggesting augmented platelet reactivity in this group. Platelet P2RY12 and SELP mRNA were increased by twofold at elevated platelet activation in contrast to control subjects. To conclude, all found miRNAs (miR-223, miR-26b, miR-126, miR-140) are down-regulated in platelets and MKs of T2DM patients, leading to higher expression of P2RY12 and SELP mRNAs, which in turn bring about changes in platelet functions [60].

Overall, the studies demonstrate that platelet miRNAs could reflect platelet function directly or indirectly, for example through their impact on mRNA processing. Many studies described in previous sections also refer to platelets and show the potential for miRNAs to become an excellent diagnostic tool covering many pathological mechanisms simultaneously. Taking into account the importance of maintaining appropriate platelet function in T2DM patients, who are a group of high cardiovascular risk, miRNAs could help repurpose existing drugs and yield new antiplatelet therapies with significant benefits in these individuals. It is worth mentioning that certain miRNAs are commonly co-expressed in diabetic environment, as evidenced in the studies conducted by Zampetaki et al. and Witkowski et al. [61, 62]. For example, miR-126 was revealed to be co-expressed with miR-19a in uncontrolled T2DM, which leads to modulation of some of the effects of miR-126, including control of VCAM1 and therefore monocyte recruitment and atherogenesis. Thus, rather than focusing on single miRNAs that are involved in T2DM an integrated view should also take into account the expression pattern of multiple miRNAs that reflect the complexity of the epigenetic regulation of vascular homeostasis in T2DM.

**Fig. 1:**
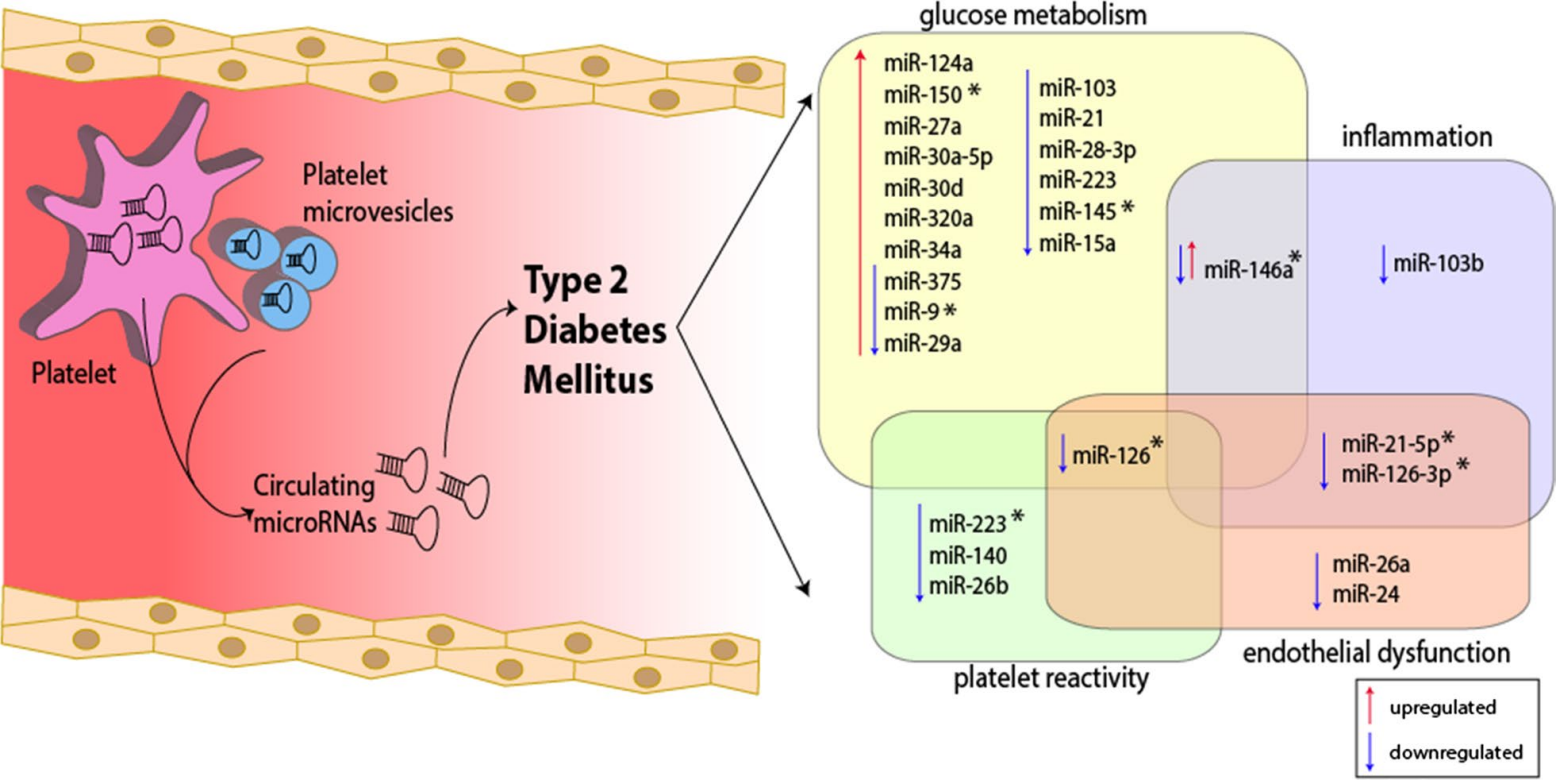
Regulation of microRNAs serving as T2DM biomarkers based on glucose metabolism, inflammation, platelet reactivity and endothelial dysfunction [36, 63–68]. *MiR* microRNA. *Endothelium-enrichment miRNAs and miRNAs involved in the regulation of endothelial cell functions

MicroRNA studies related to T2DM can be classified according to numerous criteria. Having dissected the complex pathogenesis of T2DM into several processes, we aimed to demonstrate that many mechanisms ought to be taken into account in full elucidation of T2DM and related complications (see Fig. 1). Since miRNAs can regulate more than one target gene and exert effects on several levels simultaneously, it is necessary to adopt a holistic approach on all contributing factors. Among the miRNA discussed in this review, miR-126 looks very promising, being the most studied in relation to T2DM. However, circulating levels of miR-126 are also known to the modulated in patients with CAD, acute coronary syndromes, heart failure, as well as by antiplatelet treatment [63, 69–71]. Hence, any of these conditions could represent a potential cofounder if miR-126 is used in T2DM to predict its future development.

### Bioinformatic analysis—miRNA targets predictions, data filtering and visualization as interaction networks

Coordinated interplay of inflammation, platelet activity, blood coagulation and insulin secretion is implicated in the pathogenesis of T2DM and resulting complications. Our bioinformatics analysis aimed to depict and characterize the miRNAs, which potentially contribute to the pathophysiology of increased platelet reactivity in T2DM through the mentioned mechanisms.

### Target prediction

To identify targets of analyzed miRNAs we used multiMiR 1.4 R package [72]. We searched the top 10% genes among all conserved and non-conserved target sites in 14 target prediction databases. For input miRNAs without mature version of id’s we performed target predictions using all three combinations: stem-loop miRNA and -3p and -5p versions.

### Interaction networks

MiRNA-target interaction network was constructed in R and visualized using Cytoscape software v 7 [73]. Gene–gene interaction data were retrieved from StringApp package version 1.4.2 for Cytoscape. Final networks for genes associated with analyzed processes and pathways were constructed as follows: (1) first we selected all target genes and associated with specific process. (2) Then we subtracted those genes and associated miRNAs as a new process-specific network. (3) From that network we extracted nodes connected with at least 10 other nodes (for gene it was at least 10 other genes, for miRNA at least 10 other targets).

We also performed separated unbiased analysis for all genes and input miRNAs. Due to its complexity we selected nodes with at least 20 neighbors. On all final networks we showed only max top 10 targets and max top 10 miRNAs sorted by the degree of connection. It enabled to retrieve top miRNAs and top shared targets involved in analyzed Gene Ontology (GO) process, we also preformed unbiased analysis on all of the targets of analyzed miRNAs. To construct summary figure we merged all seven of subnetworks containing miRNA-target and target interactions.

### Selection of specific lists of genes

To identify the genes associated with analyzed processes (platelet activation, blood coagulation, inflammatory response and glucose metabolism) we performed a screening of the GO terms for the presence of the key words using the biomaRt package in R [74]. Key words used for screening the GO terms are available as in our previous paper [17]. For glucose metabolism list, used GO terms were as follows: response to glucose; positive regulation of glucose metabolic process; glucose metabolic process; regulation of glucose metabolic process; glucose mediated signaling pathway; positive regulation of glucose mediated signaling pathway; UDP-glucose metabolic process To retrieve the genes associated with insulin signaling we combined gene lists from KEGG database (hsa04910 Insulin signaling pathway, hsa04930 type II diabetes mellitus, hsa04931 Insulin resistance) and Reactome database (R HSA 422356 Regulation of insulin secretion) [75, 76]. To retrieve the genes associated with diabetes we combined gene lists from KEGG database (hsa04930 type II diabetes mellitus) and disease query by String Cytoscape plugin for diabetes mellitus type 2 [77]. To retrieve genes associated with hypoglycemia we used results from disease query by String Cytoscape plugin for that term.

### Analysis results

**Fig. 2:**
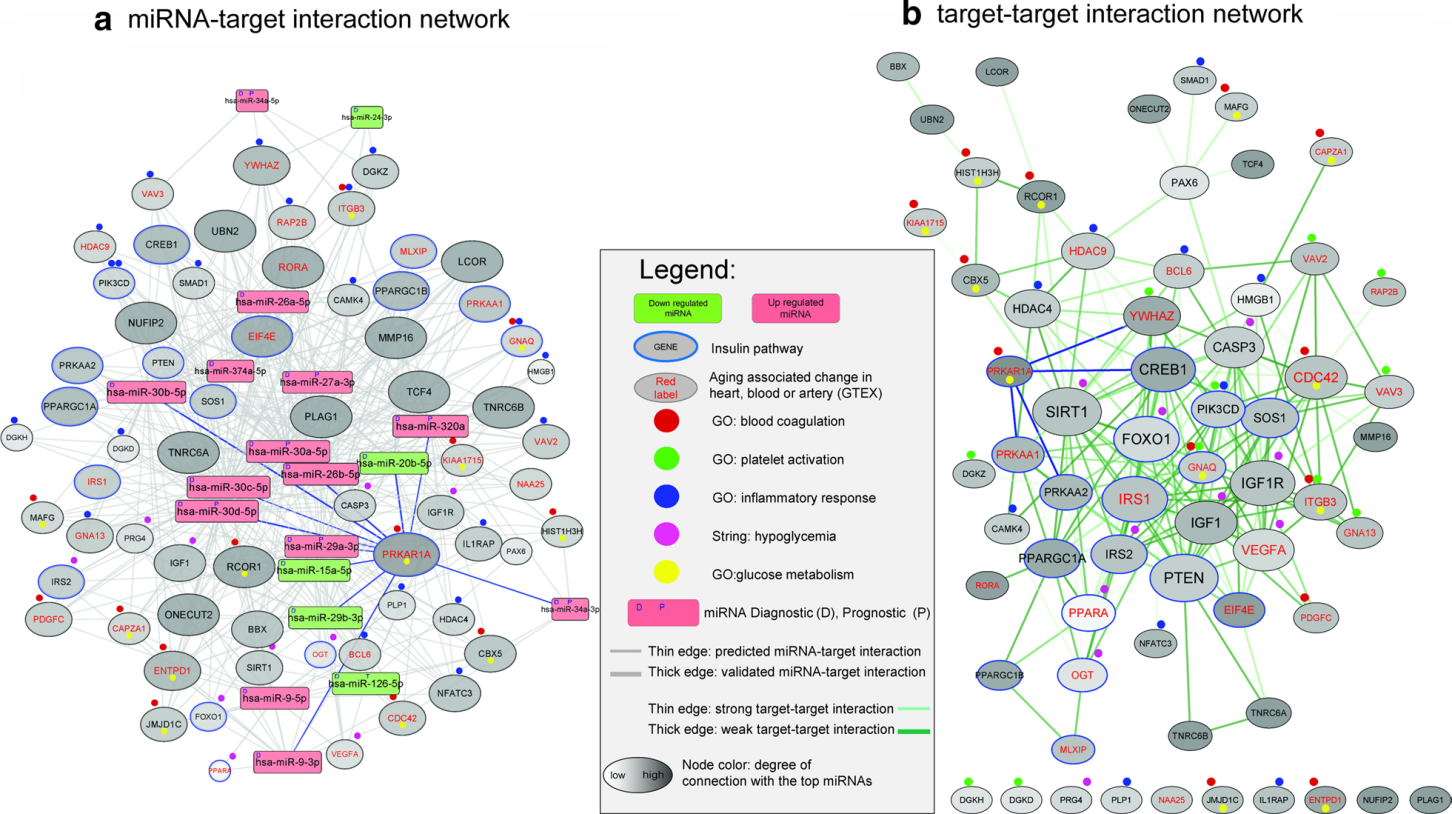
**a** MicroRNA-target interaction network, **b** Target–target interaction network. The rectangles indicate microRNAs, the ellipses indicate target genes. Red, green, blue, violet and yellow marks represent specific GO process—blood coagulation, platelet activation, inflammation response, hypoglycemia, and glucose metabolism processes, respectively. Blue borders have genes associated with insulin signalling. Size of the node is associated of connection with other nodes. Color of the targeted genes is associated with the number of the regulating them top miRNAs

Firstly, we performed interaction network analysis for between the genes associated with specific biological processes and targeting them miRNAs identified based on literature search (see Additional files 2, 3). After performing integratory analysis of those results we found that three common miRNAs, namely miR-30a-5p, miR-30d-5p and miR-30c-5p might be involved in blood coagulation, platelet activation, glucose metabolism, insulin signaling and inflammation, whereas one common miRNA, miR-320a, could regulate blood coagulation, platelet activation, insulin signaling, as well as inflammatory processes (see Fig. 2). Two common miRNAs—miR-15a-5p and miR-30b-5p—may have an influence on blood coagulation, platelet activation, insulin signaling and glucose metabolism.

To this day, multiple reports on miR-30a-5p have been published in the literature, however only one study showed a link between plasma-circulating miR-30a-5p and T2DM. In line with recent CORDIOPREV trial results, miR-30a-5p was observed to be up-regulated in T2DM subjects compared to healthy controls years before the disease manifested, suggesting its promising role in predicting T2DM occurrence. The baseline plasma levels of circulating miR-30a-5p were increased in incident-T2DM subjects in comparison to non-T2DM subjects after a median follow-up of 60 months, with intermediate values in the pre-DM and incident pre-DM patients. This study revealed that plasma miR-30a-5p, as well as miR-150, miR-15a and miR-375, are deregulated before the onset of T2DM and should be further explored as novel biomarkers [35]. MiRNA-30a-5p was also up-regulated in the sera of children with newly diagnosed type 1 diabetes mellitus in a study conducted by Nielsen et al. [78]. Finally, in vitro models demonstrated that miR-30a-5p is involved in glucotoxicity-induced beta-cell dysfunction in rodents [79]. Even though these findings have not been confirmed in human studies yet, the above-mentioned findings indicate that miR-30a-5p should be investigated in greater depth in order to elucidate its links with diabetes.

MiR-30d-5p was described so far in two studies related to T2DM. Delic et al. found that miR-30d-5p was down-regulated in urinary exosomes of microalbuminuric T2DM nephropathy patients in comparison to T2DM patients without nephropathy and healthy controls. According to the authors, lower expression of miR-30d-5p is required for TGF-β apoptosis in podocytes, which further leads to T2DM nephropathy changes [80]. In line with it, Jiang et al. reported that high d-glucose treatment decreased the viability of human AC16 cardiac cells, whereas the treatment with naringenin, a flavonoid extracted from grapefruit as well as other fruits and herbs, diminished this effect through up-regulation of miRNA-30d-5p [81]. MicroRNA microarray screening revealed that the expression of serum miR-30d-5p was significantly lower in T2DM cardiomyopathy patients in comparison to healthy controls. Cardioprotective features of naringenin and the role of miR-30d-5p in this process were further confirmed during transfection of AC16 cardiac cells with miR-30d-5p inhibitor before co-treatment with naringenin, which resulted in decreased viability of AC16 cardiac cells comparing with naringenin and high glucose cohort. The results suggested that miR-30d-5p is involved in naringenin-induced cytoprotection against high glucose-induced AC16 cardiac cells injury [81]. The described studies focus on T2DM complications rather the disease itself, however they show that miR-30d-5p deserves more attention in the next studies investigating the miRNA profile of T2DM patients. Pancreas-enriched miR-30d was also significantly up-regulated in the plasma of T2DM patients vs. healthy subjects suggesting, that miR-30d might be involved in insulin regulation processes [82]. Interestingly, miR-30d family was also revealed to be involved in systemic inflammatory response syndrome pathophysiology. It was shown to correlate positively with peroxiredoxin-1 (Prdx-1), which is secreted by immune cells during inflammation and inversely correlate with pro-inflammatory cytokines (IL-1, IL-6, IL-8, C-reactive protein) [83, 84]. Moreover, miR-30d-5p was also released by in vitro activated immune cells, which further strengthens its role in inflammatory regulation [83].

The role of miR-30c-5p has been previously described in two studies linked to diabetic diseases. Stepien et al. analyzed the levels of miR-30c-5p, among other miRNAs, between blood-derived ectosomes from 15 patients with T2DM and 15 healthy controls, and assessed their angiogenic potential. It was found that the expression of miR-30c-5p (p = 0.055), miR-20a-3p (p = 0.064) and miR-26a-5p (p = 0.075) had a tendency to increase in the ectosomes-enriched plasma of T2DM patients vs. that of control subjects. The authors also stressed the pro-angiogenic effect exerted by the miR-30 family, which could be investigated and applied in clinical practice to monitor cardiovascular complications among T2DM patients [57]. Another study aimed to explore the miRNAs expression profiles in fetal tissues of gestational diabetes mellitus (GDM) patients and normoglycaemic controls. It revealed that seven miRNAs, including miR-30c-5p, miR-452-5p, miR-126-3p, miR-130b-3p, miR-148a-3p, miR-let-7a-5p and miR-let-7g-5p, were up-regulated in the human umbilical vein endothelial cells of infants of GDM patients in comparison to controls [85].

Furthermore, in our analysis we revealed the most significant gene targeted by miRNAs linked to glucose metabolism, blood coagulation and insulin signalling, namely *PRKAR1A* (protein kinase cAMP-dependent type I regulatory subunit alpha). This gene provides instructions for production of a regulatory, type 1 alpha subunit of protein kinase A (PKA), an enzyme which promotes cell growth and proliferation [86]. Interestingly, Hussain et al. demonstrated the influence of *PRKAR1A* on the regulation of PKA-mediated pancreatic beta-cell insulin secretion. *PRKAR1A* ablation and PKA disinhibition in beta-cells in mice increased glucose-dependent insulin secretion. Furthermore, inactivating PRKAR1A mutations (Carney’s complex) in humans also resulted in oral glucose tolerance test increased insulin excursions, while plasma glucose excursion were alleviated in comparison to control subjects with similar medical history but without *PRKAR1A* mutations. The authors stressed that the humans are heterozygotes and the glucose tolerance tests were via oral administration as opposed to the intravenous administration in homozygous Δ-prkar1a mice. These differences could clarify the subtle phenotype in humans as compared to Δ-prkar1a mice [87]. Gene–gene interaction network analysis showed that *PRKAR1A* is interacting with four genes which are targets of the top miRNAs. Three of them (*CREB1*, *PRKAA1*, *PRKAA2*) are associated with insulin resistance (KEGG pathway hsa04931) and one (*YWHAZ*) was found as a top gene in platelet activation network (see Additional file 3).

## Conclusions

The increasing prevalence rates of T2DM and its complications impose serious health burden worldwide. Numerous risk factors of T2DM, silent progression of the disease until the emergence of micro- and macrovascular complications and insufficient results of the common therapies call for an urgent need to develop effective methods of early detection, new efficient therapies and prevention measures for the control of T2DM. Hypercoagulable state and the atherothrombotic sequelae are among the most important concerns in modern management of T2DM and its complications.

Since platelets are unquestionably involved in atherosclerosis, it is essential to discover their molecular background and links with T2DM. Importantly, platelets play a significant role in coordinating inflammatory and immune responses. Whereas increased platelet activity helps the organism fight infections, uncontrolled activity might result in inflammation-mediated tissue damage, as observed in T2DM [13]. Furthermore, hyperglycemia and defective glucose metabolism observed in T2DM are known to impair endothelial and platelet function, which in turn contribute to development of vascular complications and prothrombotic state [52, 88]. Cumulatively, these mechanisms provide evidence that hyperglycemia-related platelet dysfunction, as well as inflammation, might underpin the fact that diabetics are prone to developing CVDs and hence are at risk of increased morbidity and mortality. Platelets therefore are fine cellular indicators of the co-occurrence of T2DM and cardiovascular complications.

The sourceful content of miRNAs in platelets, their biochemical stability in bodily fluids, as well as relative tissue specificity indicate that they might become powerful, non-invasive biomarkers of T2DM and platelet function. The evidence of a large redundancy among the targets of the miRNAs that are modulated in T2DM is worth a comment. In fact, this is a common finding for microRNAs that exert their function as fine modulators of gene expression and work in the form of epigenetic regulation networks where multiple miRNAs contribute to multiple targets genes. Detailed analysis of regulatory networks has important clinical implications. Not only does it provide the research community with reliable information but it also represent the basis to build up complex interaction models to be used in the next future to deliver a personalized approach to diagnosis and treatment. In fact, miRNA profiling of individual patients might be analysed in the bioinformatic model to improve both diagnostic and therapeutic efficiency, especially for chronic and degenerative diseases, where the paradigm one gene, one protein, one disease does not reflect the actual pathophysiology. The studies described in this review demonstrate that various miRNA differentiate T2DM from healthy state, however we observed a discrepancy between miRNAs evaluated by researchers so far and miRNAs obtained as a result of our bioinformatic analysis. For example, miR-126, which has been investigated in most reports included in this manuscript was not deemed significant enough in the analysis. The inconsistent findings may be partly due to differences in demographic characteristics between study populations, small size of study populations and the fact that frequently more focus is placed on one pathological mechanism of T2DM, rather than a combination of several factors. Furthermore, bias in the clinical studies measuring miRNA expression may be related to the study population (T2DM vs. uncontrolled T2DM). The duration of the disease and the extend of vascular inflammation/complications certainly impacts the epigenetic environment as well). It is worth noticing that up to this day, few studies directly investigated platelet-derived miRNAs in the context of T2DM, however the involvement of platelets in many processes leading to T2DM and related complications provides a promising area for research and potential clinical application.

In this review, we described the methods currently used to detect and investigate miRNAs and summarized the studies on miRNAs in T2DM published so far. Through the application of bioinformatic analysis tools, we associated key biological function and signaling pathways related to the miRNAs with the most prominent differential expression pattern in T2DM compared to controls. Using this approach, we found three common miRNAs, namely miR-30a-5p, miR-30d-5p and miR-30c-5p as the miRNAs involved in blood coagulation, platelet activation, glucose metabolism, insulin signalling and inflammation. Additionally, results of the bioinformatics analysis demonstrate that *PRKAR1A* could be one of the most significant gene targeted by miRNAs in T2DM.

## Supplementary information

**Additional file 1.** Additional characteristics of microRNA studies in T2DM.

**Additional file 2.** MicroRNA-target gene networks a) Glucose metabolism miRNA-target gene network b) Blood coagulation miRNA-target gene network c) Hypoglycemia miRNA-target gene network d) Inflammatory response miRNA-target gene network. The rectangles indicate microRNAs, the ellipses indicate target genes. Red, green, blue, violet and yellow marks represent specific GO process—blood coagulation, platelet activation, inflammation response, hypoglycemia, and glucose metabolism processes, respectively. Blue borders have genes associated with insulin signalling. Top 4 targets are highlighted from each network with colored edges.

**Additional file 3.** MicroRNA–target gene networks. a) Insulin metabolism miRNA-target gene network. b) Platelet activation miRNA–target gene network. c) Top-unbiased. top ten gene targeted by top ten miRNAs sorted by the degree of connection. The rectangles indicate microRNAs, the ellipses indicate target genes. Red, green, blue, violet and yellow marks represent specific GO process - blood coagulation, platelet activation, inflammation response, hypoglycemia, and glucose metabolism processes, respectively. Blue borders have genes associated with insulin signalling. Top 4 targets are highlighted from each network with colored edges.

## Data Availability

Not applicable.

## Abbreviations

T2DM: type 2 diabetes mellitus
CVD: cardiovascular disease
MPs: microparticles
miRNAs: microRNAs
qRT-PCR: quantitative reverse transcription polymerase chain reaction
NGS: next-generation sequencing
NGT: normal glucose tolerance
IGT: impaired glucose tolerance
IFG: impaired fasting glucose
ROC: receiver operator characteristic
HbA1c: glycated hemoglobin
TNFs: tumour necrosis factors
IL-6: interleukin-6
NFκβ: nuclear factor κΒ
mRNA: messenger RNA
FBG: fasting blood glucose
SFRP4: secreted frizzled-related protein 4
CHD: coronary heart disease
T2DM NC: T2DM subjects without complications
T2DM C: T2DM subjects with complications
MACE: major cardiovascular events
VCAM-1: vascular cell adhesion protein-1
TF: tissue factor
CAD: coronary artery disease
AUC: area under the curve
MKs: megakaryocytes
GO: Gene Ontology
Prdx-1: peroxiredoxin-1
GDM: gestational diabetes mellitus
*PRKAR1A*: protein kinase cAMP-dependent type I regulatory subunit alpha
PKA: protein kinase A
