# Supplementary figures and images for: Significance of circulating microRNAs in diabetes mellitus type 2 and platelet reactivity: bioinformatic analysis and review
Source: Cardiovasc Diabetol. 2019 Aug 30;18:113. doi: 10.1186/s12933-019-0918-x (PMC6716825; doi:10.1186/s12933-019-0918-x)

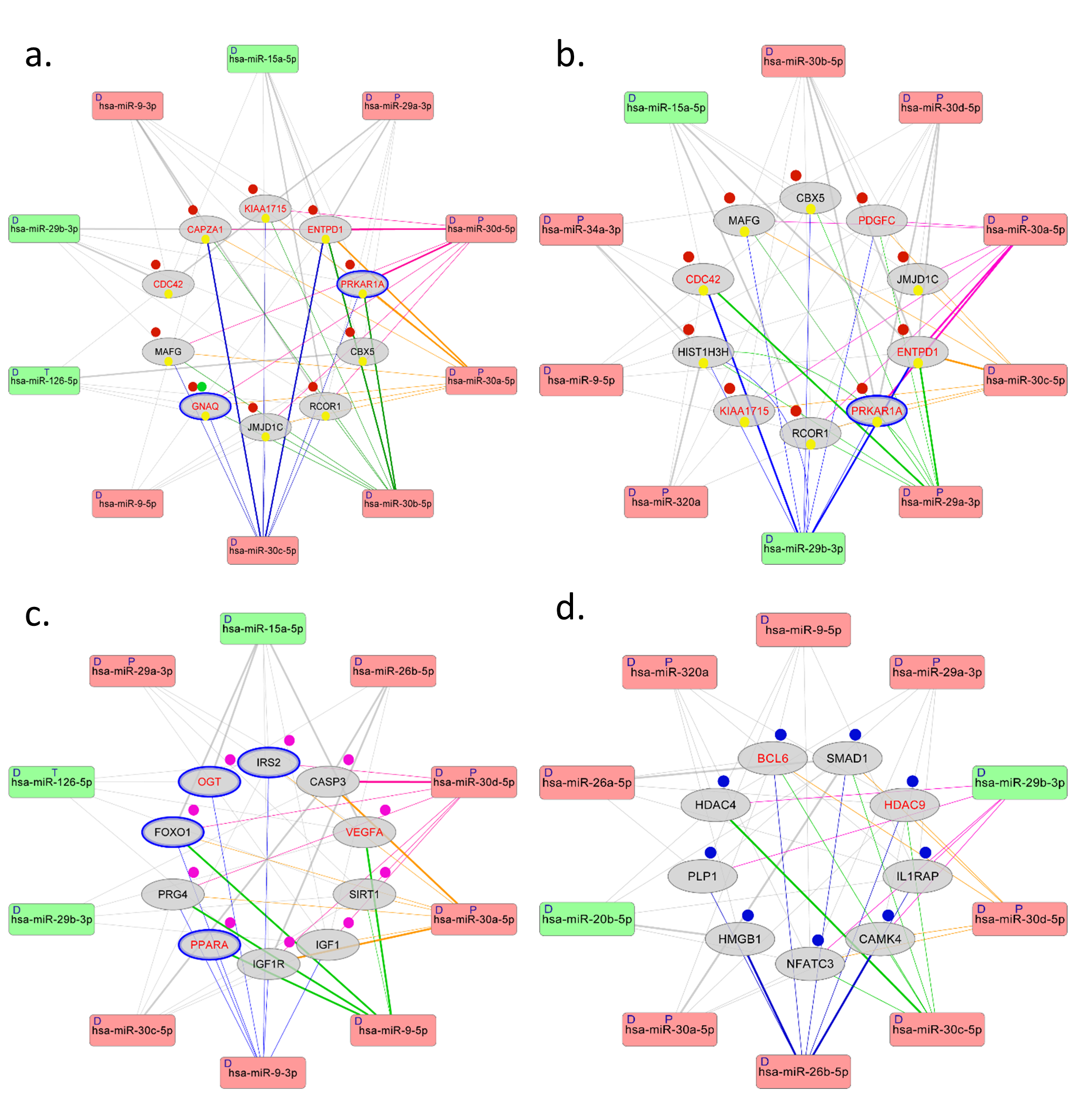

Supplement: Supplementary file 2 — Additional file 2. MicroRNA-target gene networks a) Glucose metabolism miRNA-target gene network b) Blood coagulation miRNA-target gene network c) Hypoglycemia miRNA-target gene network d) Inflammatory response miRNA-target gene network. The rectangles indicate microRNAs, the ellipses indicate target genes. Red, green, blue, violet and yellow marks represent specific GO process—blood coagulation, platelet activation, inflammation response, hypoglycemia, and glucose metabolism processes, respectively. Blue borders have genes associated with insulin signalling. Top 4 targets are highlighted from each network with colored edges. [file 12933_2019_918_MOESM2_ESM.tif]

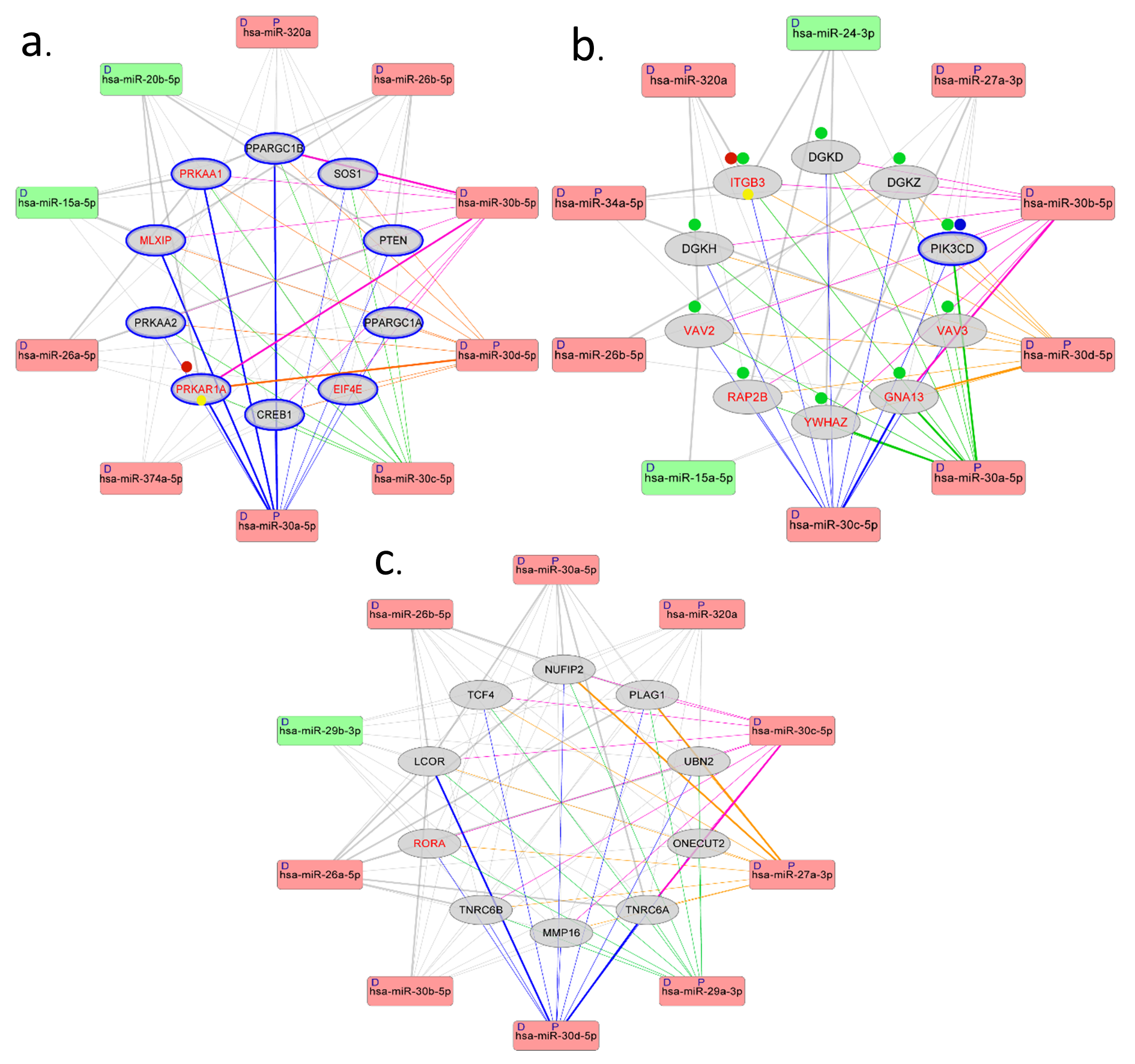

Supplement: Supplementary file 3 — Additional file 3. MicroRNA–target gene networks. a) Insulin metabolism miRNA-target gene network. b) Platelet activation miRNA–target gene network. c) Top-unbiased. top ten gene targeted by top ten miRNAs sorted by the degree of connection. The rectangles indicate microRNAs, the ellipses indicate target genes. Red, green, blue, violet and yellow marks represent specific GO process - blood coagulation, platelet activation, inflammation response, hypoglycemia, and glucose metabolism processes, respectively. Blue borders have genes associated with insulin signalling. Top 4 targets are highlighted from each network with colored edges. [file 12933_2019_918_MOESM3_ESM.tif]
